# The use of bootstrap methods for analysing health-related quality of life outcomes (particularly the SF-36)

**DOI:** 10.1186/1477-7525-2-70

**Published:** 2004-12-09

**Authors:** Stephen J Walters, Michael J Campbell

**Affiliations:** 1 Medical Statistics Group, School of Health and Related Research, University of Sheffield, UK

**Keywords:** Health Related Quality of Life, SF-36, Bootstrap Simulation, Statistical Analysis.

## Abstract

Health-Related Quality of Life (HRQoL) measures are becoming increasingly used in clinical trials as primary outcome measures. Investigators are now asking statisticians for advice on how to analyse studies that have used HRQoL outcomes.

HRQoL outcomes, like the SF-36, are usually measured on an ordinal scale. However, most investigators assume that there exists an underlying continuous latent variable that measures HRQoL, and that the actual measured outcomes (the ordered categories), reflect contiguous intervals along this continuum.

The ordinal scaling of HRQoL measures means they tend to generate data that have discrete, bounded and skewed distributions. Thus, standard methods of analysis such as the *t*-test and linear regression that assume Normality and constant variance may not be appropriate. For this reason, conventional statistical advice would suggest that non-parametric methods be used to analyse HRQoL data. The bootstrap is one such computer intensive non-parametric method for analysing data.

We used the bootstrap for hypothesis testing and the estimation of standard errors and confidence intervals for parameters, in four datasets (which illustrate the different aspects of study design). We then compared and contrasted the bootstrap with standard methods of analysing HRQoL outcomes. The standard methods included *t*-tests, linear regression, summary measures and General Linear Models.

Overall, in the datasets we studied, using the SF-36 outcome, bootstrap methods produce results similar to conventional statistical methods. This is likely because the *t*-test and linear regression are robust to the violations of assumptions that HRQoL data are likely to cause (i.e. non-Normality). While particular to our datasets, these findings are likely to generalise to other HRQoL outcomes, which have discrete, bounded and skewed distributions. Future research with other HRQoL outcome measures, interventions and populations, is required to confirm this conclusion.

## 1. Introduction

Health Related Quality of Life (HRQoL) measures are now frequently used in clinical trials and health services research, both as primary and secondary endpoints [1]. Investigators are now asking statisticians for advice on how to plan and analyse studies that have used HRQoL measures.

HRQoL measures such as the Short Form (SF)-36, Nottingham Health Profile (NHP) and European Organisation for Research and Treatment of Cancer (EORTC) QLQ-C30 are described in Fayers and Machin [1] and are usually measured on an ordered categorical (ordinal) scale. This means that responses to individual questions are usually classified into a small number of ordered response categories, e.g. poor, moderate and good. The responses are often analysed by assigning equally spaced numerical scores to the ordinal categories (e.g. 0 = 'poor', 1 = 'moderate' and 2 = 'good') and the scores across similar questions are then summed to generate a HRQoL score. These 'summated scores' are usually treated as if they were from a continuous distribution and were Normally distributed. We will also assume that there exists an underlying continuous latent variable, Z, that measures HRQoL (although not necessarily Normally distributed), and that the actual measured outcomes, X, are ordered categories that reflect contiguous intervals along this continuum.

This ordinal scaling of HRQoL measures means they generate data with discrete, bounded and non-standard distributions, which may lead to several problems in determining sample size and analysing the data [2,3]. Since HRQoL outcome measures may not meet the distributional requirements (usually that the data have a Normal distribution) for parametric methods of sample size estimation and analysis, conventional statistical advice would suggest that non-parametric methods be used to analyse HRQoL data.

The bootstrap [4,5] is a data based simulation method for estimating sample size [6] and analysing data: including hypothesis testing (p-values), standard error (SE) and confidence interval (CI) estimation; which involves repeatedly drawing random samples from the original data, with replacement. So, in theory, computer intensive methods such as the bootstrap that make no distributional assumptions may be more appropriate for estimating sample size and analysing HRQoL data than conventional statistical methods.

Conventional methods of analysis of HRQoL outcomes are extensively described in Fayers and Machin [1] and Fairclough [7]. They did not use the bootstrap to analyse HRQoL outcomes. As a consequence of this omission, the aim of this paper is to compare bootstrap computer simulation methods with standard methods of analysis of HRQoL measures (particularly the SF-36). We used the bootstrap for hypothesis testing, estimation of SEs and CIs for parameters, in four datasets (which illustrate the different aspects of study design). We then compared the bootstrap with standard methods of analysing HRQoL outcomes. These standard methods included: *t*-tests; multiple regression/analysis of covariance (ANCOVA) models fitted via ordinary least squares (OLS); response feature analysis or summary measures such as the Area Under the Curve (AUC) [8] and Generalised Linear regression Models (GLMs) [Pages 21–44, [9]] fitted using Generalised Estimating Equations (GEE) [10].

The remainder of this paper is structured into the following sections. The SF-36 HRQoL outcome is briefly described in Section 2. Section 2 also describes how the bootstrap can be used for hypothesis testing and confidence interval estimation. Section 2 ends with a description of the four example datasets. The results of conventional methods of analysis and bootstrap methods are compared in Section 3. Other issues, such as withdrawals and study sizes are discussed in Section 4. The final section (5) ends with a summary and conclusions.

## 2. Methods

### The bootstrap

The term bootstrap derives from the phrase "*to pull oneself up by one's bootstraps*". The phrase is thought to be based on one of the eighteenth century Adventures of Baron Munchausen by Rudolph Erich Raspe. The Baron had fallen to the bottom of a deep lake. Just when it looked like all was lost, he thought to pick himself up by his own bootstraps [Page 5, [4]]!

The basic idea of the bootstrap involves repeated random sampling with replacement from the original data, to produce random samples of the same size of the original sample, each of which is known as a *bootstrap sample*, and each provides an estimate of the parameter of interest, e.g. mean. The "with replacement" means that any observation can be sampled more than once in each bootstrap sample. It is important because sampling without replacement would simply give a random permutation of the original data, with many statistics such as the mean being exactly the same [Page 115, [11]]. Repeating the process a larger number of times provides the required information on the variability of the estimator, since the standard error is estimated from the standard deviation of the statistics derived from the bootstrap samples.

#### Bootstrap observed value of the test statistic

The bootstrap is mainly used as a method for assessing statistical accuracy i.e. SE, biases and CIs. Throughout this paper we shall use the observed value of the test statistic or parameter estimate as our best guess at the true value of the unknown parameter or statistic. For example, if we are interested in estimating the population mean (from a random sample) it may seem that the best estimator of the mean of the population is the mean of all the bootstrap estimates. This is turns out not to be the case as the mean of the all the bootstrap means is biased. The observed sample mean, from the original data, is always the best estimate of the population mean. The same result applies for other statistics such as the median and regression coefficients.

#### Confidence Interval estimation

Suppose we wish to calculate a 95% confidence interval for a mean HRQoL from a sample. We take a random sample, with replacement from this data, of the same size as the original sample, and calculate the mean HRQoL of the data, in this bootstrap random sample. We do this repeatedly, a large number of times, say 1000. So we now have 1000 bootstrap samples, and 1000 estimates of the sample mean, one from each bootstrap sample. If these 1000 bootstrap sample means are ordered in increasing value, a bootstrap 95% confidence interval for the mean would be from the 25^*th *^to the 975^th ^largest values. This is known as the *percentile method *and although it is an obvious choice, it is not the best method for bootstrapping confidence intervals, because it can have a bias, which one can estimate and correct for. This leads to methods such as the preferred *bias corrected and accelerated *(BC_a_) method [4,5]. Using the bootstrap method, valid bootstrap confidence intervals can be constructed for all common estimators such as the sample mean, median, proportion, difference in means, and difference in proportions. We estimated BC_a _bootstrap confidence intervals using the bootstrap procedure in STATA v8 [12].

According to Efron and Tibshirani [Page 180, [4]] each interval , where  and  are the lower and upper bounds of the interval respectively, can be described by its *length *and *shape*,



'Shape' measures the symmetry of the interval about the point estimate . The standard Normal based intervals are symmetrical about , and hence have shape = 1.00. Shape is a measure of skewness of the CI about the point estimate. A shape > 1.00, implies the CI is positively skewed, with a long tail to the right, whereas shape < 1.00 implies the CI is negatively skewed.

#### Hypothesis testing with the bootstrap

Bootstrap methods can also be used for hypothesis testing. The two quantities that we must choose when carrying out a bootstrap hypothesis test are a *test statistic *and a *null distribution for the data under the null hypothesis*. Given these, we generate bootstrap values of the test statistic under the null distribution for the data and estimate the *achieved significance level *(ASL) by calculating the proportion of the bootstrap values of the test statistics, which are greater than or equal to the observed value of the test statistic from the original data.

Several bootstrap test statistics are available for comparing the distribution of sample data in two independent groups. In considering a bootstrap hypothesis for comparing the two means, there is no compelling reason to assume equal variances and so we do not make this assumption. We used a bootstrap test statistic for comparing two means that use only the assumption of a common mean, under the null hypothesis [Page 224, [4]].

#### Linear regression: Model (residual) and case resampling

Standard errors and CIs for regression coefficients can also be obtained using bootstrap methods. Two different approaches are possible, *case *and *model (residual) *resampling.

For example with the simple linear model, *y *= *a *+ *bx*, where *y *is the outcome variable and *x *is a predictor or explanatory variable, *a *is the intercept and *b *is the slope or gradient of the line, with *n *(x, y) pairs of HRQoL observations. Then case-based resampling involves drawing a bootstrap sample of size *n*, with replacement from these *n *pairs. Ordinary least squares (OLS) are then used to estimate the regression coefficients for this bootstrap sample of paired cases. Again we do this repeatedly, say 1000 times, so we now have 1000 bootstrap samples and 1000 estimates of the regression coefficients, one from each bootstrap sample. The standard error of these estimated coefficients is simply the standard deviation of these 1000 estimates. As before we can calculate BC_a _confidence intervals for these estimated regression coefficients.

Case-based resampling may be entirely natural for situations where it is plausible that the (*x*, *y*) pairs have been drawn by random sampling from a population. However, case based resampling is less appealing if the *x *values were controlled for in some way, perhaps by the design of the study. In this situation the alternative *model or residual *based procedures could be used.

For model based resampling the conventional fitted values and residuals are first obtained from the observed data. A bootstrap sample of the residuals is then drawn. These residuals are then added to the original regression equation (and x values) to generate new bootstrap values for the outcome variable. Ordinary least squares are then used to estimate the new bootstrap regression coefficients, for this bootstrap sample. This process (resampling of the residuals, adding them to the fitted values and estimating the regression coefficients) is repeated lots of times to estimate standard errors and confidence intervals for the regression coefficients from the bootstrap samples.

Thus model based resampling is an example of the "*parametric bootstrap*" when the residuals from a parametric model are bootstrapped to give estimates of the standard error of the parameters. There is considerable debate about which form of resampling is more appropriate. Both forms of resampling can easily be implemented in STATA [12] and S-PLUS [13]. We now briefly describe the SF-36 outcome and the four example datasets.

### SF-36 Health Survey

**Figure 1:**
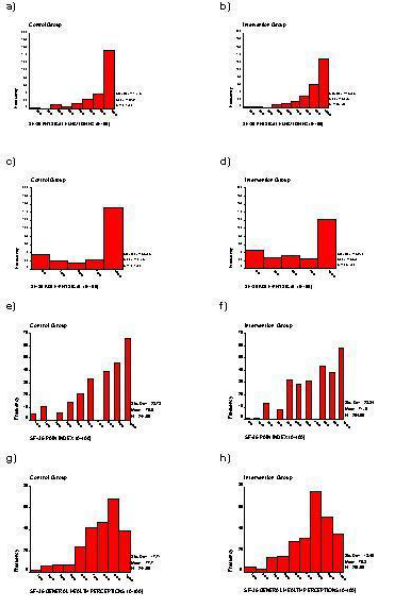
Distribution SF-36 dimensions from CPSW data by group

**Figure 2:**
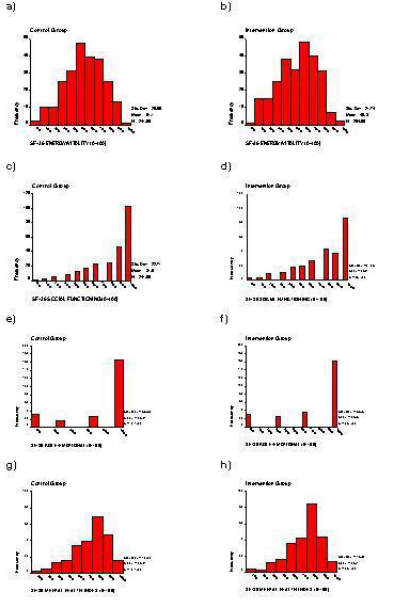
Distribution SF-36 dimensions from CPSW data by group

The SF-36 is one of the most commonly used HRQoL measures in the world today. It contains 36 questions measuring health across eight different dimensions – physical functioning (PF), role limitation because of physical health (RP), social functioning (SF), vitality (VT), bodily pain (BP), mental health (MH), role limitation because of emotional problems (RE) and general health (GH). Responses to each question within a dimension are combined to generate a score from 0 to 100, where 100 indicates "good health" [14]. Thus, the SF-36 generates a profile of HRQoL outcomes, on eight dimensions, with discrete, bounded and skewed distributions (see Figures 1 and 2) which makes statistical analysis and interpretation difficult [1].

### The four datasets

There now follows a brief description of the four datasets which are used throughout the rest of this paper. These datasets illustrate the use of HRQoL outcomes across a variety of study designs. There are three types of study: observational (both cross-sectional and with baseline and a single follow-up assessment), two group randomised controlled trial (RCT) and longitudinal RCT (with several follow-ups).

#### CPSW Data: Costs & effectiveness of community postnatal support workers (CPSW): RCT [15]

This RCT aimed to establish the relative cost-effectiveness of postnatal support in the community compared to the usual care provided by community midwives. Six hundred and twenty-three postnatal women were allocated at random to **Intervention **(n = 311) or **Control **(n = 312) groups. The intervention consisted of up to 10 home visits in the first postnatal month of up to three hours duration by a community postnatal support worker (CPSW). The main outcomes were HRQoL as measured by the SF-36 at six weeks postnatally. This study is unusual since no baseline HRQoL assessment was made. It was felt that it was inappropriate to assess HRQoL just prior to or immediately after childbirth.

Our analysis is based on the 495 responders to the six-week postnatal questionnaire who completed all 36 items of the SF-36. This sample consisted of 241 women in the Control group and 254 women in the Intervention group. We will use this data to illustrate methods for simple two group cross-sectional comparisons of HRQoL scores using conventional (e.g. *t*-test and Mann-Whitney tests) and bootstrap hypothesis tests. We will also compare standard Normal theory (*t*-test) based CIs with their bootstrap BC_a _equivalent.

#### OA Knee Data [16]

The aim of this longitudinal observational study was to evaluate two condition specific and two generic health status questionnaires for measuring HRQoL in patients with Osteoarthritis (OA) of the Knee, and offer guidance to clinicians and researchers in choosing between them. Patients were recruited from two settings, knee surgery waiting listings and rheumatology clinics. Four self-completion questionnaires including the SF-36 were sent to the subjects on two occasions 6 months apart. Two hundred and thirty patients returned the questionnaire at initial assessment, consisting of 118 patients awaiting total knee replacement (TKR) **Surgery **and 112 patients attending **Rheumatology **outpatient clinics. At the six-month follow-up assessment, 211 patients returned the questionnaire (109 and 102 in the Surgery and Rheumatology groups respectively). The data used here are based on the 211 patients returning both assessments.

Since there was a difference in the baseline HRQoL and sociodemographic characteristics (age and gender) of the Clinic and Surgery groups, we use this dataset to illustrate multiple regression/ANCOVA methods with follow-up HRQoL as the outcome variable and baseline HRQoL, age, gender and group as covariates. We compare the conventional ordinary least squares (OLS) estimates of standard error (SE) and Confidence Interval (CI) for the group regression coefficient with their bootstrap counterparts.

#### Leg Ulcer RCT data [17]

The aim of this RCT, with one year of follow-up, was to establish the relative cost-effectiveness of community leg ulcer clinics that use four layer compression bandaging versus usual care provided by district nurses. Two hundred and thirty-three patients with venous leg ulcers were allocated at random to intervention (120) or control group (113). The intervention consisted of weekly treatment with four layer bandaging in leg ulcer clinic (**Clinic **group) or usual care at home by the district nursing service (**Home **group). The primary outcome was time to complete ulcer healing over the one-year follow-up. Secondary outcomes included HRQoL as measured by the SF-36 at baseline, three months and 12 months follow-up.

We use these data to illustrate the use of summary measures such as the AUC for analysing longitudinal data, using conventional and bootstrap hypothesis tests. We will also compare standard Normal theory (*t*-test) based CIs with their bootstrap BC_a _equivalent.

#### Early Rheumatoid Arthritis RCT data [18]

The Early Rheumatoid Arthritis or NAMEIT trial was a 48-week, randomised, double blind study to compare Neoral with methotrexate (**Neoral**) versus placebo plus methotrexate (**Placebo**) in patients with early severe rheumatoid arthritis (RA). The primary efficacy variable in this study was the attainment of American College of Rheumatology (ACR) criteria for improvement of rheumatoid arthritis. Secondary efficacy variables included patient assessment of health related quality of life (HRQoL).

In order to assess the impact of the treatments on patients' health related quality of life, the SF-36 was completed by subjects at seven time-points, Week 0 (baseline), Weeks 8, 16, 24, 32, 40, and Week 48 at the end of the study or at the time of premature withdrawal from the trial.

Three hundred and six subjects at 48 centres were actually entered into the study. One hundred and fifty-two subjects receiving methotrexate were randomised to the Neoral treatment group and 154 subjects receiving methotrexate were randomised to the Placebo group. Of the 306 subjects randomised, 227 completed the study. Seventy-nine randomised subjects discontinued from the study prior to completion.

We use these data to illustrate more complex statistical models for analysing longitudinal data e.g. a marginal GLM fitted with GEEs and compare bootstrap SEs and CIs for the parameters with their conventionally estimated counterparts.

## 3. Results

### Dataset 1 CPSW Study: simple cross-sectional comparison of 6 week HRQoL for the Control vs. Intervention Groups

Figures 1 and 2 show the histograms of the SF-36 dimension scores at six weeks post-natally for Intervention and Control groups. The graphs clearly show the bounded, skewed and discrete nature of the data for the SF-36 from this study.

Table 1 shows the two sample *t*-test (with equal variances) and Mann-Whitney (MW) comparisons of the eight SF-36 dimension scores. If we assume a cut-off of p ≤ 0.05 for statistical significance, then the *t*-test suggests significant differences on two dimensions of the SF-36: RP and SF. On two other dimensions PF (p = 0.060) and BP (p = 0.065) the *p*-values are close to the arbitrary cut-off of 0.05, suggesting some differences although these may not be statistically reliable. The results of the *MW *tests suggest significant differences on four dimensions (PF, RP, BP and SF) of the SF-36. The only major contrast between the interpretation of the results of the *MW *and *t*-tests is on the BP and PF dimensions, where the former test suggests a difference and later not.

**Table 1:** CPSW Study Simple cross-sectional comparison of 6 week HRQoL for Control vs. Intervention Groups

***SF-36 Dimension***	***Group***	***n***	***mean***	***sd***	***Mean Diff***	***t-test equal σ's P-value***	***MW test P-value***	***Bootstrap P-value***
*Physical*	Control	241	89.9	14.5	2.6	0.060	0.015	0.057
*Function*	Intervention	254	87.3	15.8				
*Role*	Control	241	74.3	38.1	9.1	0.009	0.004	0.010
*Physical*	Intervention	254	65.2	39.5				
*Bodily*	Control	241	75.6	23.7	4	0.065	0.040	0.062
*Pain*	Intervention	254	71.6	23.8				
*General*	Control	241	77.7	17.7	2.4	0.139	0.147	0.131
*Health*	Intervention	254	75.3	18.5				
*Vitality*	Control	241	51.1	20.7	1.3	0.498	0.596	0.494
	Intervention	254	49.8	21.7				
*Social*	Control	241	81.6	22.7	4.7	0.025	0.015	0.024
*Function*	Intervention	254	76.9	24.2				
*Role*	Control	241	77.9	36.4	1.1	0.734	0.503	0.737
*Emotional*	Intervention	254	76.8	35.5				
*Mental*	Control	241	72.9	17.2	-0.2	0.902	0.972	0.904
*Health*	Intervention	254	73.1	16.7				

The bootstrap p-value is based on 5000 bootstrap replications.

The last column of Table 1 also shows the results of a bootstrap hypothesis test for comparing two means. It compares and contrasts the results of the *p*-values from a bootstrap hypothesis tests with the *p*-values from the standard two sample *t*-test with equal variances, and the *MW *test. Although they report quantitatively different *p*-values, the magnitudes are similar, and if we use a cut-off of p < 0.05 for statistical significance then the qualitative interpretation of the tests is the same. So in this example dataset there appears to be little advantage in using the bootstrap hypothesis tests compared to conventional hypothesis tests, such as the *t-*test, for testing equality of means.

A major limitation of non-parametric methods, such as the *MW *test, is that they do not allow for the estimation of confidence intervals for parameters or allow for the adjustment of confounding variables such as baseline covariates. One way to estimate non-parametric CIs is via the bootstrap method. Table 2 compares and contrasts the Normal/*t*-test (equal variances) based confidence intervals with the bootstrap BC_a _ones.

**Table 2:** Comparisons of parametric and bootstrap estimates of confidence intervals for the eight dimensions of the SF-36 from the CPSW Study for Control vs. Intervention Groups

***SF-36***			***CIs***		***Interval***	
***Dimension***		***Mean Difference ***	***Lower ***	***Upper ***	***Length***	***Shape***
*Physical*	Normal (*t*-test)	-2.6	-5.2	0.1	5.4	1.00
*Function*	Bootstrap BC_A_		-5.2	0.0	5.2	0.98
*Role*	Normal (*t*-test)	-9.1	-16.0	-2.3	13.7	1.00
*Physical*	Bootstrap BC_A_		-15.8	-2.3	13.5	1.02
*Bodily*	Normal (*t*-test)	-4.0	-8.2	0.2	8.4	1.00
*Pain*	Bootstrap BC_A_		-8.1	0.3	8.4	1.03
*General*	Normal (*t*-test)	-2.4	-5.6	0.8	6.4	1.00
*Health*	Bootstrap BC_A_		-5.6	0.8	6.4	0.99
*Vitality*	Normal (*t*-test)	-1.3	-5.0	2.5	7.5	1.00
	Bootstrap BC_A_		-5.1	2.4	7.5	0.98
*Social*	Normal (*t*-test)	-4.7	-8.9	-0.6	8.3	1.00
*Function*	Bootstrap BC_A_		-8.7	-0.6	8.1	1.03
*Role*	Normal (*t*-test)	-1.1	-7.5	5.3	12.7	1.00
*Emotional*	Bootstrap BC_A_		-7.1	5.6	12.7	1.11
*Mental*	Normal (*t*-test)	0.2	-2.8	3.2	6.0	1.00
*Health*	Bootstrap BC_A_		-2.8	3.2	6.0	0.98

*Mean difference * = *Intervention mean - Control mean*

*BCa confidence intervals based 5000 bootstrap replications*.

The estimates and lengths of the CIs are almost identical. Table 2 also shows that the shape of the BC_a _CIs is almost symmetric about the point estimate of the mean difference except for the RE dimension, where there is some evidence of asymmetry. So again in this example dataset there appears little advantage in using the bootstrap BC_a _confidence intervals compared to conventional methods of confidence interval estimation.

The bootstrap (and Normal) confidence intervals are calculated for a characteristic of the distributions (for example mean difference). The groups may have differences in distributions but similar characteristics e.g. mean [15]. For example, the *MW *tests suggests a significant difference (in distributions) for the PF, RP, BP and SF dimensions, but the bootstrap and Normal confidence limits for two out of four of these dimensions (PF and BP) includes zero; suggesting no differences in the mean HRQoL between the groups.

When a hypothesis is tested using the bootstrap, the resampling is carried out assuming the null hypothesis H_0 _is true. Whereas when confidence intervals for mean differences between two groups are estimated the resampling is carried out separately for each group. A useful analogy is with the comparison of proportions in two independent groups. Here the standard error for the hypothesis test is different to the standard error of the difference between the observed proportions used for estimating a confidence interval [Page 45, [19]].

### Dataset 2 OA Knee: comparison of OLS multiple regression, bootstrap case and model based resampling SE and CI estimates for the group (surgery vs. clinic) parameter

Table 3 shows the baseline socio-demographic and HRQoL characteristics of the two groups of OA patients those awaiting total knee replacement surgery (Surgical) and those having pharmacological treatment (Rheumatology). The group of patients awaiting surgery is significantly older and has significantly more men than the Rheumatology group. The Surgical group has significantly lower levels of PF prior to total knee replacement surgery than the Rheumatology group. Conversely the Surgical group has significantly higher levels of GH, V and MH compared to the Rheumatology clinic patients. For the other four dimensions of the SF-36 (RP, BP, SF and RE) there was no evidence of any difference in HRQoL between the two groups.

**Table 3:** Baseline characteristics of the TKR Surgery and Rheumatology Clinic patients from the OA Knee study.

	**Rheumatology**			**Surgical**				**95% CI**		
	**N**	**Mean**	**SD**	**N**	**Mean**	**SD**	**Mean Diff**	**Lower**	**Upper**	**P-value**
***Age (years)***	102	64.2	(11.3)	109	71.1	(8.5)	-6.9	-9.6	-4.2	0.001
***SF-36 Dimensions***										
*Physical Function*	97	28.2	(22.4)	95	21.2	(18.2)	7.0	1.2	12.8	0.019
*Role Physical*	96	11.5	(22.0)	99	12.9	(26.3)	-1.4	-8.3	5.4	0.684
*Bodily Pain*	100	32.0	(19.5)	104	36.3	(23.4)	-4.3	-10.3	1.6	0.154
*General Health*	94	43.9	(22.9)	96	57.3	(23.8)	-13.3	-20.0	-6.6	0.001
*Vitality*	98	36.9	(19.0)	99	42.3	(19.3)	-5.4	-10.8	0.0	0.050
*Social Function*	100	53.1	(30.6)	101	53.6	(27.6)	-0.5	-8.6	7.6	0.910
*Role Emotional*	95	41.1	(44.2)	99	44.1	(44.6)	-3.1	-15.6	9.5	0.632
*Mental Health*	99	62.7	(20.9)	100	68.2	(18.8)	-5.5	-11.0	0.1	0.054
***Gender***										
*Female*	71		(69.6%)	59		(54.1%)	(15.5%)	(2.4%)	(27.8%)	0.021†
*Male*	31		(30.4%)	50		(45.9%)				
*Total*	102		(100%)	109		(100%)				

*P-values from two independent samples t-test except (†) χ^2^test*.

We were interested in seeing whether or not there was a difference in HRQoL in OA patients after TKR surgery compared with pharmacologically treated patients. From previous studies using the SF-36 we know that HRQoL varies with age and gender [14,20]. Since there was a difference in the baseline HRQoL and socio-demographic characteristics (age and gender) of the Rheumatology clinic and TKR surgery groups, we use this dataset to illustrate multiple regression/ANCOVA methods with follow-up HRQoL as the outcome variable and baseline HRQoL, age, gender and group (TKR surgery or Rheumatology clinic) as covariates.

The analysis involved using OLS to fit the multiple regression model with six month follow-up HRQoL as the outcome variable and age in years at baseline; gender of the patient (coded 0 for males and 1 for females); baseline HRQoL and treatment group variable (coded 0 = Clinic, 1 = Surgery) as explanatory covariates. The group regression coefficient estimate represents the difference in six-month follow-up HRQoL between the Rheumatology Clinic and TKR Surgery groups after adjustment for the patient's age, gender and baseline HRQoL. A positive value for the regression coefficient indicates the Surgery group has a better mean HRQoL at six months follow-up than the Clinic group after adjustment for the other covariates.

Table 4 compares the OLS and bootstrap standard errors and confidence interval estimates for the group coefficient from the OA Knee data. All models include age, baseline HRQoL and gender as covariates in the regression. For the bootstrap methods the standard errors are the standard deviations of the coefficients from the 5000 bootstrap re-samples. For ease of interpretation and comparison only the estimates for the group coefficient are shown.

**Table 4:** Comparison of multiple regression, bootstrap case and model based resampling SE and CI estimates from the OA Knee data

**Dependent**		**GROUP coefficient**					**95% CI**		**Interval**	
**Variable**	**Model**	**N**		**SE**	/*SE*	**p**	**Lower**	**Upper**	**Length**	**Shape**
**Physical Function**	OLS	165	**13.3**	3.07	4.31	0.001	7.19	19.32	12.14	1.00
	Case			3.02	4.39		7.64	19.69	12.05	1.15
	Model			3.05	4.35		7.49	19.49	12.00	1.08
**Role Physical**	OLS	177	**-0.5**	4.89	-0.11	0.915	-10.16	9.12	19.29	1.00
	Case			4.39	-0.12		-8.60	8.51	17.11	1.12
	Model			4.86	-0.11		-10.11	8.93	19.04	0.99
**Bodily Pain**	OLS	200	**14.7**	3.39	4.34	0.000	8.01	21.38	13.37	1.00
	Case			3.41	4.30		7.81	21.41	13.60	0.98
	Model			3.36	4.38		8.07	21.25	13.18	0.99
**General Health**	OLS	173	**4.7**	2.01	2.32	0.021	0.71	8.65	7.95	1.00
	Case			2.03	7.26		0.69	8.69	8.00	1.01
	Model			1.98	2.37		0.70	8.48	7.78	0.95
**Energy**	OLS	185	**6.5**	2.46	2.64	0.009	1.65	11.36	9.72	1.00
	Case			2.50	2.60		1.75	11.63	9.88	1.08
	Model			2.46	2.65		1.49	11.04	9.54	0.90
**Social Function**	OLS	194	**9.1**	3.70	2.46	0.015	1.82	16.41	14.59	1.00
	Case			3.52	2.59		2.14	16.06	13.92	1.00
	Model			3.65	2.50		1.72	16.09	14.37	0.94
**Role Emotional**	OLS	184	**9.4**	6.10	1.55	0.124	-2.60	21.48	24.08	1.00
	Case			5.89	1.60		-1.90	20.85	22.75	1.01
	Model			6.03	1.57		-2.37	20.90	23.27	0.97
**Mental Health**	OLS	191	**1.1**	2.15	0.51	0.613	-3.15	5.33	8.48	1.00
	Case			2.32	0.47		-3.54	5.42	8.96	0.94
	Model			2.14	0.51		-3.17	5.12	8.28	0.95

*Bootstrap BCa confidence intervals based 5000 bootstrap replications*.

The regression analysis suggests that at six month follow-up TKR surgical patients have significantly better HRQoL than Rheumatology treated clinic patients on five dimensions of the SF-36 (PF, BP, GH, V and SF) after adjustment for age, gender and baseline HRQoL. As can be seen from Table 4 the standard error estimates are almost identical for the three methods. Similarly the length of the confidence intervals is virtually the same for all three methods. Although the bootstrap CIs tend to be asymmetric about the point-estimate of the regression coefficient.

Qualitatively all of the intervals from the three methods either include or exclude zero so the interpretation of the group regression coefficient is the same. Therefore, again in this example dataset, there appears to be little advantage in using bootstrap case or model based re-sampling to estimate standard errors and confidence intervals compared to conventional methods of confidence interval estimation from the OLS multiple regression model.

### Dataset 3 Leg ulcer: simple cross-sectional comparison of AUC for Home vs. Clinic Groups

We are interested in comparing the HRQoL over the one-year follow-up between the Home and Clinic treated groups. The two groups were well matched at baseline for age, gender and HRQoL, except for the RE dimension of the SF-36, where there was some reliable statistical evidence of a difference (p = 0.052).

The overall HRQoL of the leg ulcer patients over the 12-month study period (and three HRQoL assessments) can be summarised by the AUC. If we set the time units for the AUC calculation as a fraction of a year, then an AUC value of 100 implies the leg ulcer patient has been in "good health" for the entire 12-month follow-up period. Conversely an AUC value of 0 implies the leg ulcer patient has been in "poor health" for the entire 12-month follow-up period.

Table 7 [See additional file 1] gives the results of simple comparisons of differences in mean AUC between the groups using the two independent samples *t*-test, the *MW *test and the bootstrap hypothesis test.

The *p*-values from the *t*-test and the ASL from the bootstrap hypothesis tests are very similar. None of the *p*-values for the eight SF-36 dimensions are less than 0.05. Therefore there is no reliable statistical evidence to suggest a difference in mean AUC between the Clinic and Home treated leg-ulcer patients. Only the results of the *MW *test on the RE dimension of the SF-36 provide (p = 0.071) any evidence of a difference in AUC distributions between the groups, although even this *p*-value is not statistically significant using the conventional cut-off of 0.05.

The table also contrasts the Normal theory based CI estimates from the *t*-test with the bootstrap BC_a _limits. The lengths of the intervals are very similar, although the bootstrap BC_a _intervals tend to have a non-symmetric shape. All the estimated CIs include zero, again suggesting no evidence of a difference in mean AUC (HRQoL) between the Clinic and Home group patients in the Leg Ulcer study.

### Dataset 4 Early RA: Comparison of robust and bootstrap SE's and CI's for the time and group coefficients with a GEE marginal model and exchangeable autocorrelation

In the Early RA study, HRQoL assessment was carried out at 0, 8, 16, 24, 32, 40 and 48 weeks. With seven repeated HRQoL measurements, such as this, the best approach is to model the longitudinal data using GLMs.

The modelling of longitudinal data takes into account the fact that successive HRQoL assessments by a particular subject are likely to be correlated. We used a marginal model with the Early RA data and used GEEs to estimate the regression coefficients. Marginal models are appropriate when inferences about the population average are the focus. For example, in a clinical trial the average difference between control and treatment is most important, not the difference for any one individual. In a marginal model, the regression of the response on explanatory variables is modelled separately from the within-person correlation.

The marginal model is an extension of the linear regression model used with the OA Knee data. Longitudinal models require the specification of the *auto- *or *serial correlation*, which is the strength of the association between successive longitudinal measurements of a single HRQoL variable on the same patient.

Several underlying patterns of the auto-correlation matrix are used in the modelling of HRQoL data. The error structure is *independent *(sometimes termed *random*) if the off diagonal terms of the auto-correlation matrix are zero. The repeated HRQoL observations on the same subject are then independent of each other, and can be regarded as though they were observations from different individuals. On the other hand, if all the correlations are approximately equal or *uniform *then the matrix of correlation coefficients is termed *exchangeable*, or *compound symmetric*. This means that we can re-order (exchange) the successive observations in any way we choose in our data file without affecting the pattern in the correlation matrix. As the time or lag between successive observations increases, the auto-correlation between the observations decreases. A correlation matrix of this form is said to have an *autoregressive structure *(sometimes called *multiplicative *or *time series*).

Table 5 summarises the resulting 21 auto-correlation pairs for the assessments until week 48. The pattern of the observed auto-correlation matrix, gives a guide to the so-called error structure associated with the successive HRQoL measurements. Table 5 shows that the autocorrelation coefficients range between 0.19 and 0.85. For three dimensions of the SF-36, PF, GH and MH, the autocorrelation coefficients are moderately large (between 0.5 and 0.85). The pattern of values suggests that the assumption of compound symmetry is not unreasonable.

**Table 5:** Auto-correlation matrices for the eight dimensions of the SF-36 from RA patients in the Early RA study assessed at seven time points

**a) Physical Function (n = 218)**								**e) Vitality (n = 216)**							
**Week**	**0**	**8**	**16**	**24**	**32**	**40**	**48**	**Week**	**0**	**8**	**16**	**24**	**32**	**40**	**48**
**0**	1.00							**0**	1.00						
**8**	0.61	1.00						**8**	0.55	1.00					
**16**	0.63	0.74	1.00					**16**	0.48	0.58	1.00				
**24**	0.57	0.69	0.75	1.00				**24**	0.47	0.54	0.71	1.00			
**32**	0.56	0.68	0.80	0.79	1.00			**32**	0.50	0.59	0.68	0.71	1.00		
**40**	0.55	0.67	0.77	0.81	0.86	1.00		**40**	0.42	0.49	0.67	0.68	0.77	1.00	
**48**	0.53	0.64	0.74	0.81	0.81	0.85	^1.00^	**48**	0.47	0.53	0.66	0.72	0.72	0.76	^1.00^
**b) Role Physical (n = 212)**								**f) Social Function (n = 219)**							
**Week**	**0**	**8**	**16**	**24**	**32**	**40**	**48**	**Week**	**0**	**8**	**16**	**24**	**32**	**40**	**48**
**0**	1.00							**0**	1.00						
**8**	0.40	1.00						**8**	0.44	1.00					
**16**	0.35	0.53	1.00					**16**	0.43	0.53	1.00				
**24**	0.29	0.39	0.57	1.00				**24**	0.39	0.55	0.63	1.00			
**32**	0.19	0.30	0.56	0.67	1.00			**32**	0.36	0.46	0.63	0.70	1.00		
**40**	0.34	0.42	0.52	0.60	0.61	1.00		**40**	0.38	0.51	0.58	0.64	0.71	1.00	
**48**	0.27	0.40	0.59	0.67	0.64	0.71	^1.00^	**48**	0.34	0.45	0.58	0.64	0.71	0.71	^1.00^
**c) Bodily Pain (n = 219)**								**g) Role Emotional (n = 206)**							
**Week**	**0**	**8**	**16**	**24**	**32**	**40**	**48**	**Week**	**0**	**8**	**16**	**24**	**32**	**40**	**48**
**0**	1.00							**0**	1.00						
**8**	0.43	1.00						**8**	0.46	1.00					
**16**	0.45	0.55	1.00					**16**	0.35	0.47	1.00				
**24**	0.44	0.47	0.61	1.00				**24**	0.34	0.40	0.59	1.00			
**32**	0.37	0.46	0.51	0.68	1.00			**32**	0.31	0.32	0.56	0.62	1.00		
**40**	0.40	0.42	0.57	0.60	0.69	1.00		**40**	0.34	0.46	0.53	0.56	0.54	1.00	
**48**	0.42	0.46	0.59	0.63	0.68	0.76	^1.00^	**48**	0.31	0.37	0.49	0.58	0.54	0.69	1.00
**d) General Health (n = 209)**								**h) Mental Health (n = 218)**							
**Week**	**0**	**8**	**16**	**24**	**32**	**40**	**48**	**Week**	**0**	**8**	**16**	**24**	**32**	**40**	**48**
**0**	1.00							**0**	1.00						
**8**	0.55	1.00						**8**	0.57	1.00					
**16**	0.56	0.68	1.00					**16**	0.57	0.62	1.00				
**24**	0.60	0.67	0.80	1.00				**24**	0.55	0.59	0.72	1.00			
**32**	0.58	0.67	0.77	0.83	1.00			**32**	0.52	0.55	0.65	0.69	1.00		
**40**	0.59	0.65	0.72	0.79	0.84	1.00		**40**	0.50	0.54	0.70	0.70	0.74	1.00	
**48**	0.58	0.65	0.75	0.84	0.82	0.85	^1.00^	**48**	0.56	0.55	0.68	0.72	0.73	0.77	1.00

*Correlations are Pearson's product moment coefficient*.

The process of fitting marginal models using GEE begins by assuming the simple independence form for the autocorrelation matrix, and fitting the model as if each assessment were from a different patient. Once this model is obtained the corresponding residuals are calculated and these are then used to estimate the autocorrelation matrix assuming it is of the exchangeable (or autoregressive) type. This matrix is then used to fit the model again, the residuals are once more calculated, and the autocorrelation matrix obtained. The iteration process is repeated until the corresponding regression coefficients that are obtained in the successive models converge or differ little on successive occasions [1].

**Figure 3:**
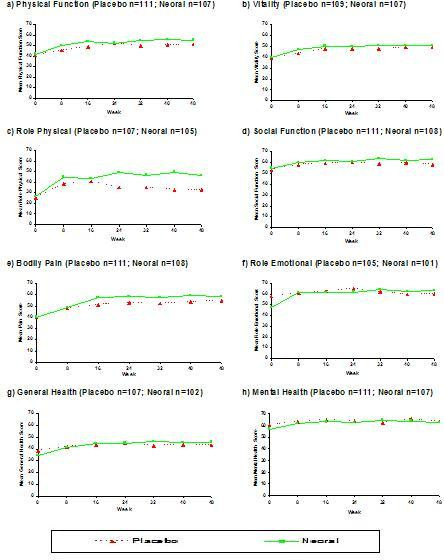
Profile of mean SF-36 scores over time by treatment group EARLY RA data (Patients who completed all seven HRQoL assessments)

Fayers and Machin [Pages 183–202, [1]] and Diggle et al [10] emphasise the importance of graphical presentation of longitudinal data prior to modelling. Figure 3 shows the mean levels of HRQoL in patients with RA, before and during treatment, for the eight dimensions of the SF-36. The curves for some dimensions of the SF-36 overlap (e.g. PF, GH, RE, and MH dimensions) suggesting that it may be unrealistic to assume that the mean difference in HRQoL values on these dimensions remains constant over time. For other dimensions such as BP, V and SF there is some evidence to suggest that for later HRQoL measurements the curves are parallel and that the mean difference between treatments is now fairly constant.

The overlapping lines on some of the graphs in Figure 3 imply there may be a 'Treatment × Time' interaction. It is therefore important to test for any such interaction in any regression model. Fortunately, with the marginal model approach this is relatively easy to do and simply involves the addition of an extra regression coefficient to the model. If treatment is coded as a 0/1 variable (i.e. 0 = Placebo and 1 = Neoral) and assessment time as a continuous variable, then the additional interaction term is simply the product of these two variables (which will be 0 for all the Placebo group patients and equal to the HRQoL assessment time in the Neoral Group patients).

#### Early RA marginal model analysis

The marginal model we used for the Early RA data for analysing the seven HRQoL assessments over time was,

*Y*_*ij *_= *β*_1 _+ *β*_*Base*_*x*_*Base_i *_+ *β*_*Age*_*x*_*Age_i *_+ *β*_*Sex*_*x*_*Sex_i *_+ *β*_*Time*_*t*_*ij *_+ *β*_*Group*_*x*_*Group_i *_+ *ε*_*ij*_,     (2)

where *Y*_*ij *_is the HRQoL at time *t*_*ij *_post-baseline; *t*_*ij *_is the time of the QoL assessment, in weeks post baseline, of patient *i *at visit *j*; *x*_*Base_i *_is the baseline HRQoL assessment for subject *i*; *x*_*Age_i *_is the age (in years) of subject *i *at time 0 (baseline); *x*_*Sex_i *_is the gender of subject *i*; *x*_*Group_i *_is the treatment group (0 = Placebo, 1 = Neoral) for subject *i*; *β*_1 _is a constant and *ε*_*ij *_is the residual error.

The marginal regression models were fitted in STATA [12] using the xtgee command with an identity link function (link (iden)) and the robust standard errors option. The observed correlation matrices in Table 5 clearly show the off-diagonal terms are non-zero and that the assumption of an independent auto-correlation matrix for the marginal model is unrealistic. We will not consider models with an independent auto-correlation structure and will concentrate on reporting the results of models with an exchangeable correlation.

None of the interaction term coefficients for the eight SF-36 dimensions were statistically significant (from zero). Thus there was no reliable evidence of a 'Treatment × Time' interaction on any dimension of the SF-36 (p > 0.05), irrespective of the autocorrelation structure. Therefore we will only report the results of the simpler model (2), without the interaction term.

The beauty of the marginal model and the GEE methodology is that it is very flexible and can in principle deal with all the observed data from a HRQoL study. The subjects are not required to have exactly the same numbers of assessments, and even the assessments can be made at variable times. The latter allows the modelling to proceed even if a subject misses a HRQoL assessment. So it seems unrealistic and unreasonable to use bootstrap resampling methods for marginal models that can only utilise a balanced data set, with equally spaced QoL assessments. Since we are interested in fitting a marginal model and we are likely to have an unbalanced dataset with unequal observations per subject we used simple bootstrap case-resampling.

**Figure 4:**
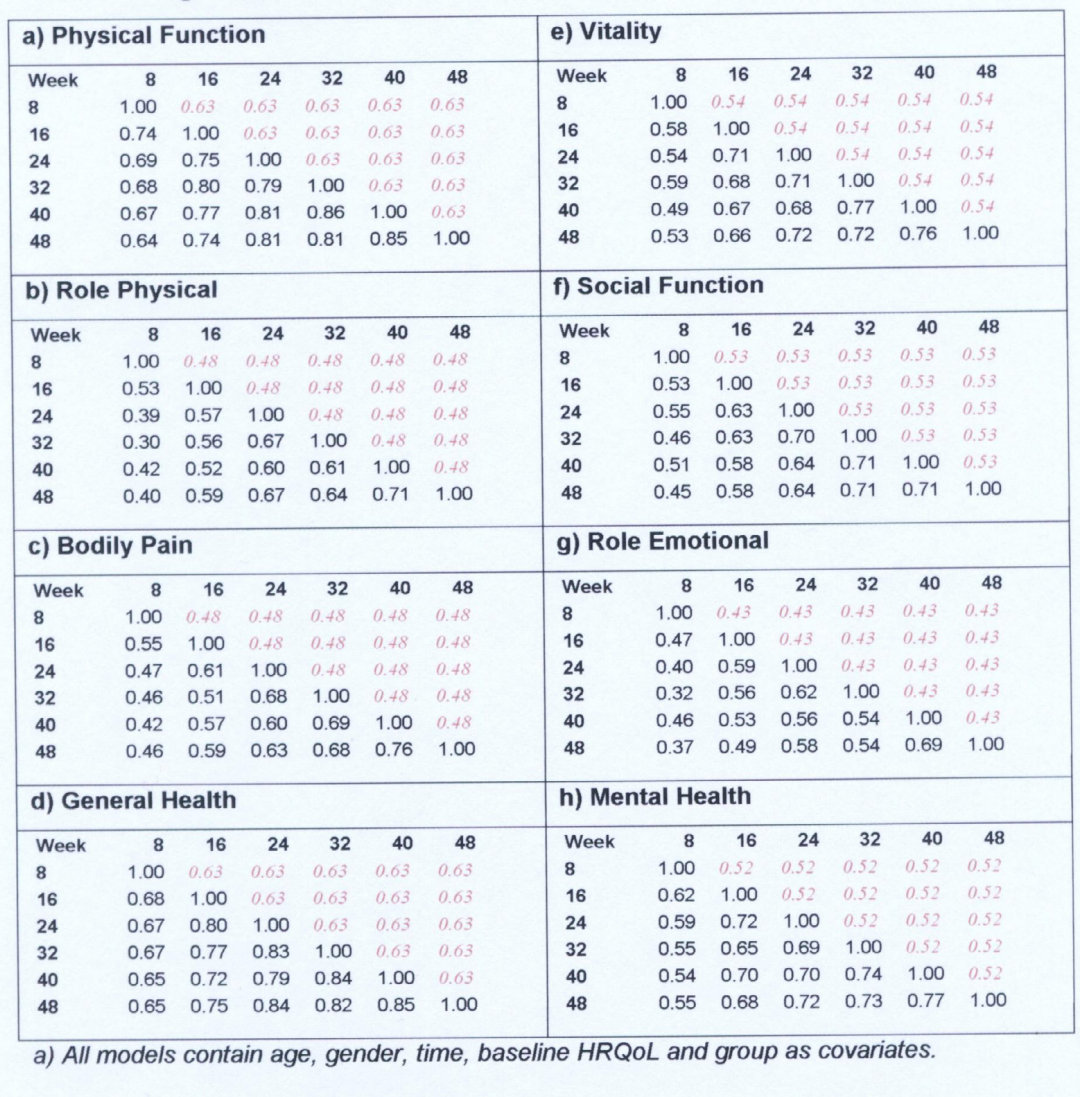
Observed and estimated within-patient auto-correlation matrices (exchangeable model) from RA patients in the EARLY RA study. The lower diagonal gives the observed matrix before model fitting whilst the upper gives the exchangeable form after model-fitting^a^

Figure 4 shows the estimated within subject correlation matrices for the eight dimensions of the SF-36 if we fit the longitudinal model and assume a compound symmetric structure. The lower diagonal gives the observed matrix before the model fitting. The fitted autocorrelations ranged from 0.43 for the RE dimension to 0.63 for the PF and GH dimensions. On the whole, the model correlation estimates tend to be lower than the actual observed autocorrelations, for HRQoL assessments that are close together. Conversely the model correlation estimates tend to be larger than the observed correlations for HRQoL observations further apart in time. It will usually be the case that after model fitting the autocorrelations will appear to have been reduced [1]. The observed deviations between the fitted model and observed autocorrelations are not too great, suggesting that the assumption of compound symmetry is not unreasonable (Figure 4).

Table 6 shows the estimated regression coefficients for the group and time variables. There is some evidence that HRQoL increases over time for three dimensions of the SF-36, PF, BP and V. However, we are interested in the effect of treatment and comparing HRQoL over time across the Placebo and Neoral treated groups. Since there is no reliable evidence of a 'Group × Time' interaction the interpretation of the treatment group coefficient is relatively straightforward. The *p*-values for the treatment group regression coefficients in Table 6 suggest significant differences in HRQoL between the Neoral and Placebo groups on three dimensions of the SF-36 (RP, GH and BP).

**Table 6:** Comparison of robust and bootstrap SE's and CI's from the EARLY RA data with a Marginal Model and exchangeable autocorrelation

**Dependent**		**Coefficients**				**95% CI**		**Interval**	
**Variable**			**SE**	/*SE*	**p**	**Lower**	**Upper**	**Length**	**Shape**
**Physical Function (n = 222)**	**time**	0.11	0.03	3.63	0.001	0.05	0.18	0.12	1.00
			*0.03*	*3.72*		*0.05*	*0.18*	*0.12*	*0.97*
	**group**	2.82	2.25	1.25	0.211	-1.60	7.24	8.84	1.00
			*1.72*	*1.64*		*-0.51*	*6.13*	*6.64*	*0.99*
**Role Physical (n = 221)**	**time**	-0.06	0.07	-0.90	0.366	-0.19	0.07	0.26	1.00
			*0.07*	*-0.94*		*-0.19*	*0.06*	*0.25*	*0.91*
	**group**	9.49	3.93	2.42	0.016	1.79	17.19	15.40	1.00
			*3.22*	*2.95*		*3.63*	*16.62*	*12.99*	*1.22*
**Bodily Pain (n = 222)**	**time**	0.16	0.03	4.69	0.001	0.10	0.23	0.14	1.00
			*0.03*	*4.88*		*0.10*	*0.23*	*0.13*	*1.05*
	**group**	4.23	1.97	2.14	0.032	0.36	8.10	7.74	1.00
			*1.50*	*2.82*		*1.44*	*7.25*	*5.81*	*1.08*
**General Health (n = 221)**	**time**	0.04	0.03	1.67	0.095	-0.01	0.09	0.10	1.00
			*0.03*	*1.68*		*-0.01*	*0.09*	*0.10*	*0.95*
	**group**	-4.61	1.96	-2.35	0.019	-8.46	-0.76	7.69	1.00
			*1.51*	*-3.04*		*-7.36*	*-1.28*	*6.07*	*1.21*
**Vitality (n = 220)**	**time**	0.09	0.03	3.09	0.002	0.03	0.14	0.11	1.00
			*0.03*	*3.05*		*0.04*	*0.15*	*0.11*	*1.24*
	**group**	2.67	1.80	1.48	0.14	-0.87	6.20	7.07	1.00
			*1.41*	*1.89*		*-0.19*	*5.42*	*5.61*	*0.96*
**Social Function (n = 222)**	**time**	0.03	0.03	0.77	0.442	-0.04	0.09	0.13	1.00
			*0.03*	*0.79*		*-0.04*	*0.09*	*0.13*	*0.97*
	**group**	2.40	2.11	1.14	0.255	-1.73	6.54	8.27	1.00
			*1.65*	*1.46*		*-0.72*	*5.88*	*6.61*	*1.11*
**Role Emotional (n = 221)**	**time**	-0.02	0.07	-0.32	0.752	-0.17	0.12	0.29	1.00
			*0.08*	*-0.31*		*-0.18*	*0.12*	*0.30*	*0.93*
	**group**	4.14	3.91	1.06	0.29	-3.52	11.81	15.33	1.00
			*2.93*	*1.41*		*-1.54*	*10.11*	*11.64*	*1.05*
**Mental Health (n = 221)**	**time**	0.02	0.03	0.69	0.489	-0.03	0.07	0.10	1.00
			*0.03*	*0.68*		*-0.03*	*0.07*	*0.10*	*1.15*
	**group**	1.53	1.67	0.92	0.359	-1.74	4.79	6.53	1.00
			*1.34*	*1.14*		*-0.93*	*4.42*	*5.35*	*1.18*

*Note: The bootstrap estimates of SE and BC_a _Confidence Intervals are shown in italics below the model based estimates and are based on 1000 resamples*.

The bootstrap and robust standard errors for the time and group coefficients are different, although the bootstrap SE estimate tends to be the same size or somewhat smaller than its robust counterpart. However both bootstrap and robust SE estimates are of a similar order of magnitude. More importantly, the ratios of the estimated coefficient to its standard error are of similar size.

A crude test of statistical significance is to examine this ratio, if it is bigger than 2.0 then the estimated regression coefficient is likely to be significantly different from zero. Table 6 shows that for all the models where the original (group or time) regression estimates are significant (i.e. ratios of estimate/SE > 2) then so too is the ratio of the estimate to its bootstrap standard error.

When we compare the bootstrap BC_a _confidence intervals with the model- based estimates in Table 6 then the length of the bootstrap intervals tend to be the same size or slightly narrower than its robust counterpart. As before the bootstrap estimates are not constrained to be symmetric about the point-estimate of the regression coefficient. Qualitatively both the bootstrap and model based intervals include zero when the estimated regression coefficient is non-significant and exclude zero when the estimated coefficient is significant. Therefore, the actual practical interpretation of the confidence interval estimates is the same. That is for the RP, BP, and GH dimensions there is some evidence that the Neoral group has a better HRQoL than the Placebo group patients over time, after allowing for baseline HRQoL, age and gender.

The use of the bootstrap to estimate SEs and CIs for marginal longitudinal models appears to offer little advantage (in the Early RA data) compared to the conventional robust estimates.

## 4. Discussion

In the datasets and outcomes studied, and for the specific conventional analyses we used, we have shown that use of the bootstrap does not lead to different p-values, SE and CI estimates compared to conventional methods. On this basis, we cannot conclude the use of the bootstrap is more appropriate than conventional methods. The explanation for this conclusion and the extent of its generalisability deserve discussion.

### Ordinality of HRQoL outcomes

One of the fundamental assumptions we have made, is that there exists an underlying continuous latent variable that measures HRQoL, and that the actual measured outcomes are ordered categories that reflect contiguous intervals along this continuum. If the goal of the analysis is to assess the magnitude of the treatment effect on this ordered outcome, then an appealing approach is to assign numeric scores to the ordered categories and then to compare means between groups using conventional linear regression methods. If interest lies elsewhere, for example in comparing the relative frequencies of cumulative probabilities in the ordered categories between treatments, then other techniques such as the proportional odds model would be more appropriate [9,2,21]. Heeren and D'Agostino [26] have demonstrated the robustness of the two independent samples *t*-test when applied to three-, four- and five point ordinal scaled data using assigned scores, in sample sizes as small as 20 subjects per group. Sullivan and D'Agostino [27] have expanded this work to account for a covariate when the outcome is ordinal in nature. They again assign numeric scores to the distinct response categories and compare means between treatment groups adjusting for a covariate reflecting a baseline assessment measured on the same scale. Their simulation study shows that in the presence of three-, four- and five point ordinal data and small sample sizes (as low as 20 per group) that both ANCOVA and the two independent sample *t*-test on difference scores are robust and produce actual significance levels close to the nominal significance levels.

### Generalisability

The generalisability of the results could be called into question as they only apply to the limited number of datasets studied (four) and the SF-36 outcome. The SF-36 outcome is the most widely used generic HRQoL measure in the world today, so that is one obvious reason to use it [22]. Secondly, we had easy access to a variety of datasets that had previously used the SF-36 outcome. The four studies (CPSW, OA Knee, Leg Ulcer and Early RA), and datasets were well known to us. They illustrate the use of HRQoL outcomes across a variety of studies including cross-sectional surveys, RCTs, non-randomised before and after studies and longitudinal designs. So on practical and pragmatic grounds, we felt it was appropriate to use such datasets because of their familiar nature and the analysis was easy to understand and interpret.

The SF-36 is a multi-dimensional outcome with eight dimensions. As described in the Introduction the eight dimensions have a variety of distributions. We believe these distributions are not atypical of other generic HRQoL measures such as the NHP and EORTC QLQ-C30. The distributions we considered were chosen based on our experiences with HRQoL data in a variety of settings. So we believe that our results about the bootstrap may have generalisability to other HRQoL outcomes (besides the SF-36) used in other studies and populations, although strictly speaking our results only apply to the SF-36 outcome and the observed datasets. Hence, we cannot make sweeping generalisations about the impact of the bootstrap on other HRQoL outcomes, used in other studies. Therefore, these results need to be replicated with other HRQoL measures in other datasets and populations.

### Missing values

It should be noted that in the all four example datasets there is missing data. We assumed that any missing HRQoL values in these datasets were Missing Completely at Random (MCAR). This means that the probability of the HRQoL response being missing is independent of the scores on the previous observed questionnaires and independent of the current and future scores had they been observed. We have assumed that the reduced dataset represents a randomly drawn sub-sample of the full dataset and the inferences drawn can be considered reasonable. This is a strong assumption and unlikely to hold for missing HRQoL data [1,7,23-25].

### Sample sizes of the example datasets

The various datasets used in this study all had a sample size in excess of 100 patients. Some caution should be used in applying the results to smaller sample sizes. However the robustness of the conventional two-sample *t*-test and ANCOVA, for three-, four- and five point ordinal scale data using assigned scores has been demonstrated for sample sizes as small as 20 [26,27]. Simple bootstrapping may not be very successful in small samples anyway (say < 9 observations), since the observations themselves are less likely to be representative of the study population. As Campbell [Page 118, [11]] states, *"In very small samples even a badly fitting parametric analysis may outperform a non-parametric analysis, by providing less variable results at the expense of a tolerable amount of bias."*

### The bootstrap

#### Bootstrap case resampling vs. model based resampling

The results with the OA Knee data show that there is little to choose from between the case and model based resampling for the multiple linear regression model for estimating SEs and CIs. Since there was very little difference in the SE and CI estimates from the datasets used, for simplicity one would tend to favour a case based resampling approach. Indeed this was the resampling method for the longitudinal marginal model for the Early RA data.

#### Bootstrap model based resampling for marginal model

In the longitudinal Early RA for simplicity we used only a simple case based resampling for the marginal model and effectively carried out a stratified random resampling with replacement. That is we sampled with replacement blocks or clusters of each patients' repeated HRQoL responses. In theory, one should be able to use model or residual based resampling for the marginal model. The resampling procedure would be rather complex particularly for autoregressive autocorrelation structures and for unbalanced datasets, with HRQoL assessments at unequally spaced time points. One would have to take into account that the residuals were not independent and uncorrelated, and for the autoregressive correlation structure, that the correlation between residuals within a patient declined over time. This is a very interesting avenue and requires further exploration with other longitudinal datasets.

### Are the results surprising or unexpected?

Finally, are the results all that surprising or unexpected? We have shown that the use of bootstrap methods for analysis (calculation of p-values, SE and CIs) appears to offer little advantage compared to standard methods in the four datasets studied.

If we assume that there exists an underlying continuous latent variable that quantifies the HRQoL response of interest and that the goal of the analysis is to assess the magnitude of a treatment effect on the HRQoL outcome, by comparing means between groups. Then statistical theory says that if the distribution of the HRQoL data is Normal, so will be the distribution of the sample mean. Much more importantly, even if the distribution of HRQoL data is not Normal, as is frequently the case, that of the sample mean will become closer to a Normal distribution as the sample size gets larger. This is a consequence of the Central Limit Theorem (CLT) [Pages 304-7, [28]]. The Normal distribution is strictly only the limiting form of the sampling distribution as the sample size increases to infinity, but it provides a remarkable good approximation to the sampling distribution even when the sample size is small and the distribution of the data is far from Normal [Page 94, [29]]. This implies, for example, that the distribution of the sample means for the SF-36 HRQoL data shown in Figures 1 and 2 will be approximately Normal.

Thus, if the investigator is planning a large study and the sample mean is an appropriate summary measure of the HRQoL outcome, then pragmatically there is no need to worry about the distribution of the HRQoL outcome and we can use standard methods to estimate sample sizes and analyse the data. Since dramatic effects are unlikely in HRQoL studies using the SF-36 as an outcome, large samples sizes are likely to be required [2,3,6]. So perhaps unsurprisingly, the results reflect the robustness of conventional methods with large sample sizes and the application of the CLT to sample means even for HRQoL data with such bounded, discrete and skewed distributions as shown in Figures 1 and 2.

So our research using the SF-36 HRQoL outcome and the four datasets has shown that bootstrap methods appear to produce p-values, SEs and CIs similar to conventional methods. When the standard and the bootstrap methods agree, we can be more confident about the inference we are making and this is an important use of the bootstrap [Page 118, [11]]. When they disagree more caution is needed, but the relatively simple assumptions required by the bootstrap method for validity mean that in general it is to be preferred. Thus, there appears to be little advantage in using the bootstrap for the analysis of SF-36 data, particularly if one is interested in comparing mean HRQoL between treatment groups.

## 5. Conclusions

In the datasets we studied, using the SF-36 as an outcome measure, bootstrap methods produce results similar to conventional statistical methods. This is likely because the *t*-test and OLS multiple regression are robust to the violations of assumptions that HRQoL data are likely to cause (i.e. non-Normality). While particular to our datasets, these findings are likely to generalise to other HRQoL outcomes, which have discrete, bounded and skewed distributions. They may not generalise to HRQoL studies with smaller sample sizes of less than 100 subjects. Future research with other HRQoL outcome measures, interventions and populations, is required to confirm this conclusion.

## Supplementary Material

Additional File 1 Table 7 – Leg Ulcer study simple cross-sectional comparison of AUC for Home vs. Clinic Groups Click here for file
