# Supplementary figures and images for: The use of bootstrap methods for analysing health-related quality of life outcomes (particularly the SF-36)
Source: Health Qual Life Outcomes. 2004 Dec 9;2:70. doi: 10.1186/1477-7525-2-70 (PMC543443; doi:10.1186/1477-7525-2-70)

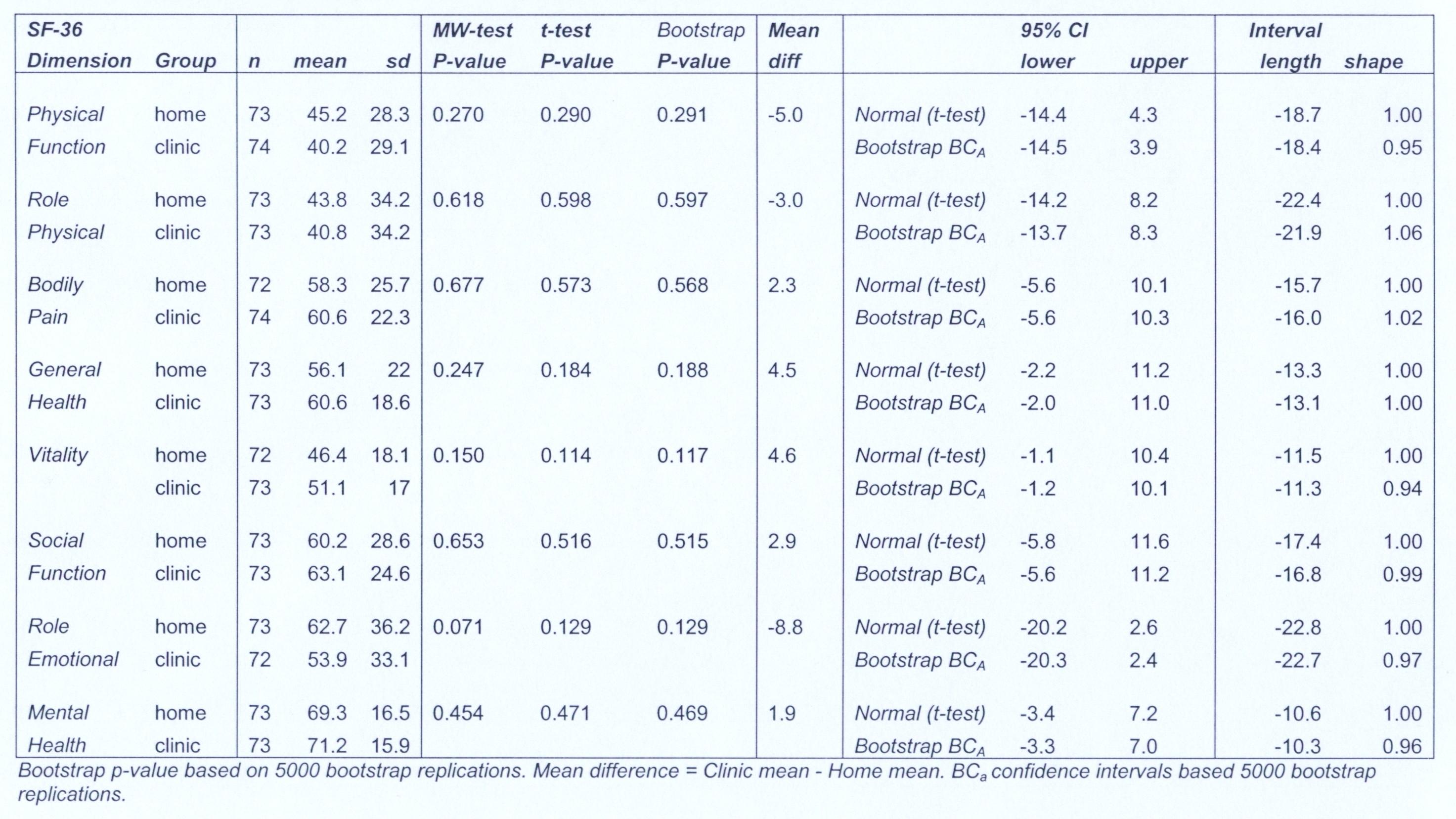

Supplement: Additional File 1 — Table 7 – Leg Ulcer study simple cross-sectional comparison of AUC for Home vs. Clinic Groups [file 1477-7525-2-70-S1.jpeg]
